# Salvianolic acid B inhibits mitochondrial dysfunction by up-regulating mortalin

**DOI:** 10.1038/srep43097

**Published:** 2017-03-02

**Authors:** Yunxia Liu, Yingying Hu, Qiukai E, Ji Zuo, Ling Yang, Wen Liu

**Affiliations:** 1 Department of Cellular and Genetic Medicine, School of Basic Medical Sciences, Fudan University, Shanghai 200032, China; 2 Pathophysiology Department, Key Laboratory of Cell Differentiation and Apoptosis of Chinese Ministry of Education, Shanghai Jiaotong University, Shanghai 200025, P.R. China; 3 Obstetrics and Gynecology Hospital of Fudan University, Shanghai 200011, China; 4 Department of Pathology, Shanghai Shuguang Hospital affiliated with Shanghai University of Traditional Chinese Medicine, Shanghai 201203, China

## Abstract

Salvianolic acid B is an antioxidative ingredient derived from Radix *Salviae miltiorrhizae* that has been widely used to treat liver diseases. However, the therapeutic mechanism underlying Salvianolic acid B has remained largely unknown. Our studies verified that Salvianolic acid B efficiently blocked mitochondrial deformation and dysfunction induced by H_2_O_2_ in the human hepatocyte cell line HL7702. Mortalin, a mitochondrial molecular chaperone, maintains mitochondrial morphology stabilization and function integrity. Previous results showed that mortalin overexpression has been observed in hematoma carcinoma cells and that mortalin maintains mitochondrial homeostasis and antagonizes oxidative stress damage. We found that Salvianolic acid B significantly up-regulated mortalin protein expression levels. In addition, Salvianolic acid B lost the function of preventing mitochondrial deformation and dysfunction induced by oxidative stress under mortalin knockdown conditions. We further found that mortalin overexpression increases the mRNA expression of mitofusin-related factor Mfn1 and mitofission-related factor hFis1. In conclusion, Salvianolic acid B maintains the mitochondrial structure stabilization and functional integrity by up-regulating mortalin, which may be associated with increased mitofusin factor Mfn1 and reduced mitofission factor hFis1.

Hepatic disease is a major global health problem with significant morbidity and increased mortality that affects one hundred million people worldwide. Hepatic disease is characterized by additional collagen and accumulation of extracellular matrix in response to hepatocellular damage^1^. Clinical and experimental evidence suggest that oxidative stress and mitochondrial dysfunction mediated the pathological progression of hepatocellular damage^2^,^3^. Numerous studies have found that reactive oxygen species (ROS) play a critical role in producing liver damage and hepatic disease by stimulating the generation of profibrogenic mediators from Kupffer cells and directly stimulating hepatic satellite cells (HSC)^4^. Therefore, exploring novel antioxidants that prevent hepatic fibrogenesis is one of the most important strategies in treating liver diseases.

Salvianolic acid B (SalB, C_36_H_30_O_16_, molecular weight = 718.6138 g/mol) is the aqueous bioactive component from *Salvia miltiorrhiza* bunge. SalB is a polyphenlic compound found in abundance in this plant^5^. SalB is among the most effective natural antioxidants and has significant scavenging effects on oxygen free radicals and protecting the liver against injury and fibrosis^6^,^7^,^8^. Previous studies reported that SalB is effective in inhibiting hepatocellular apoptosis by regulating apoptotic-related factors in the mitochondrial death pathway. SalB is also involved in ameliorating mitochondrial energy metabolism^9^,^10^. In living cells, mitochondria are relevant sources of reactive oxygen species. Werner J.H Koopman found that redox homeostasis is closely associated with mitochondrial morphology and function^11^. Mitochondria are dynamic organelles that are present in virtually every mammalian cell and display a continuous cycle of fission and fusion^12^,^13^. Mitochondrial fission is guided by Dynamic-related protein 1(Drp1) and human fission protein1 (hFis1), whereas mitochondrial fusion is executed by two mitofusins (Mfn1/Mfn2) and optic atrophy 1 protein (Opa1)^14^,^15^. However, the mechanisms of action for SalB on hepatocyte apoptosis have not been thoroughly elucidated, especially those associated with mitochondrial morphology and function.

Mortalin (mtHsp70/Hsp75/Grp75/PBP74) is a heat un-inducible protein that is a heat shock protein 7 (Hsp70) family member. Mortalin is located predominantly in mitochondria, where it functions to maintain mitochondrial homeostasis and quality control^16^,^17^. As an important mitochondrial chaperone, mortalin drives nuclear-encoded proteins across the mitochondrial membrane into the matrix by an ATP-dependent mechanism via the interaction of other chaperones^18^,^19^. Previous studies show that mortalin overexpression inhibits cell apoptosis by attenuating/reducing accumulation of ROS during various forms of stresses^20^,^21^. Mortalin knockdown is also effective in mitochondrial dynamics. Mortalin induces mitochondrial fission by regulating the balance of mitochondrial dynamics^22^.

Therefore, this study primarily focuses on investigating the effects of SalB on mitochondrial homeostasis and the balance of mitochondrial dynamics and testing whether mortalin can protect against mitochondrial dysfunction by regulating the balance of mitochondrial dynamics under H_2_O_2_-induced oxidative stress. Additionally, we studied the possible mechanisms of SalB mitochondrial protection by assessing the correlation with mortalin.

## Materials and Methods

### Materials

HL-7702 Cells were purchased from the Institute of Biochemistry and Cell Biology, China Academy of Science, Shanghai. Salvianolic acid B, which was obtained from Traditional Chinese Medicine of Shanghai University, was dissolved in sterile distilled water. In addition, 3-(4,5-dimethylthiazol-2-yl)-2,5-diphenyltetrazolium bromide (MTT) was purchased from Sigma-Aldrich (St. Louis, MO, USA). Dimethylsulfoxide (DMSO) was obtained from Shenggong Biology (Shanghai, China). Mitochondrial membrane potential detection kit, ATP detection kit and Hoechst 33258 were purchased from Beyotime (Jiangsu, China). Dulbecco’s modified Eagle’s medium (DMEM) supplement was obtained from Gibco Invitrogen Co. (Gaithersburg, MD, USA). The fluorescent dye 2′,7′-dichlorodihydro-fluorescein diacetate (H2DCF-DA) and MitoTrackerGreen (MTG) were purchased from Invitrogen (Gaithersburg, MD, USA). Antibodies against cytochrome c, caspase 3 and mortalin were obtained from Cell Signaling Technology (Beverly, MA, USA) and Epitomics (Burlingame, CA, USA). Antibodies against Bcl-2 and Bax were purchased from Epitomics (Burlingame, CA, USA), and antibodies against Mfn2 and hFis1were acquired from Proteintech (Proteintech, Rosemont, IL, USA). All the other chemicals were of the highest grade of purity available commercially.

### Cell culture and treatment

HL-7702 cells were routinely cultured in DMEM (Invitrogen, Gaithersburg, MD, USA) containing 10% fetal calf serum (FBS; Biowest, Caille, France), 100 U/ml penicillin and 100 mg/ml streptomycin and maintained at 37 °C with 5% CO_2_. Cells were incubated with 400 μM H_2_O_2_ for 2 h to induce cell apoptosis and pre-incubated with various concentrations of SalB for 2 h.

### Preparation of SalB stock solution

The stock solution of SalB was prepared by dissolving 10 mg SalB using 1.39156 ml sterile distilled water to generate a 10 mM solution that was stored at −70 °C for future use.

### Plasmid construction

To construct the mortalin plasmid, the mortalin target sequence was prepared from HL-7702 cellular cDNA and subcloned into pcDNA3.1(+). The construct was verified by sequencing. The primers used in the experiments were 5′-GGTAC CTTTATCCGCCATGATAAGTGCCAG-3′ (sense) and 5′-GGATCCTCAGGAAGT CTCTTCACTCCTAAG-3′ (anti-sense).

### Lentivirus-meditated RNA interference

Lentivirus-meditated RNA interference was performed as previously described [16]. HL-7702 cells were infected with a lentivirus expressing shRNA targeting the CGTGCTCAATTTGAAGGGATT sequence of mortalin. This sequence was assessed against the BLAST database to confirm specificity. A scrambled shRNA sequence was used as a control.

### Determination of cell viability

Cell viability was measured by conventional MTT reduction assay. The cultured cells were seeded at an initial density of 5 × 10^4 ^cells/ml in a 96-well plate for 24 h and pre-incubated with 50, 100 and 200 μM SalB for 2 h and exposed to 400 μM H_2_O_2_ for 2 h. Following incubation, a 20-μl MTT stock solution (5 mg/ml) was added to each well at a final concentration of 0.5 mg/ml for an additional 4 h. The resulting formazan was dissolved in 150 μl DMSO and measured with a microtiter plate reader (Thermo Fisher Scientific, Waltham, MA, USA) at a wavelength of 492 nm.

### Intracellular ROS detection

Intracellular ROS was detected by H2DCF-DA, an oxidation-sensitive fluorescent probe. Normal and mortalin knockdown (KD) cells were pre-incubated with SalB for 2 h and exposed to 400 μM H_2_O_2_ for 2 h. The medium was removed. Cells were washed twice with serum-free medium and incubated with H2DCF-DA (10 μM) for 20 min at 37 °C in the dark. The fluorescence intensity was monitored on an automatic fluorescence microplate reader with an excitation wavelength of 488 nm and an emission wavelength of 525 nm and was observed under a DM2000 fluorescence microscope (Leica Microsystems, Wetzlar, Germany) equipped with a Leica DFC420 camera.

### Detection of ATP levels

Intracellular ATP levels were measured by a firefly-luciferase-based ATP detection kit (Beyotime, Jiangsu, China) according to the manufacturer’s instructions. Briefly, cells were seeded into the 24-well plates and washed with phosphate-buffered saline (PBS) thrice. The supernatant of samples was collected immediately on ice and measured with an illuminometer. ATP levels were calculated according to an ATP standard curve. Intracellular ATP levels were analyzed as the percentage of the control group.

### MMP measurement

Intracellular MMP was evaluated using the fluorescent, lipophilic and cationic probe, 5,5′,6,6′-tetrachloro-1,1′,3,3′-iodide (JC-1) (Beyotime, Jiangsu, China) according to the manufacturer’s instructions. Briefly, following treatment, the cells were loaded with JC-1 staining solution for 20 min at 37 °C and washed thrice with JC-1 staining buffer. The fluorescence images were obtained with a DM2000 fluorescence microscope equipped with a Leica DFC420 camera. The fluorescence intensity was measured using a CytoFluor multiwell plate reader at 514 nm for excitation and 529 nm for emission of green (monomer form) fluorescence and 585 nm for excitation and 590 nm for emission for red (aggregate form) fluorescence. The MMP of cells in each group was evaluated as the fluorescence ratio of red to green and observed using a fluorescence microscope.

### Immunofluorescence

Cells or mortalin knockdown cells were placed on cover slips in 24-well plates and pretreated with SalB for 2 h prior to exposure to 400 μM H_2_O_2_ for 2 h. After washing with PBS, the slides were fixed in 4% paraformaldehyde for 10 min at room temperature, washed thrice with PBS, permeabilized with 0.1% saponin, blocked with 10% normal goat serum and incubated overnight at 4 °C with cytochrome c antibody. The slides were incubated with FITC-conjugated goat anti-rabbit immunoglobulin (Sigma-Aldrich, Burlingame, CA, USA) for 2 h. Nuclei were stained with Hoechst 33258 (Beyotime, Jiangsu, China). Cover slips were observed under a fluorescence microscope (Leica Microsystems, Wetzlar, Germany) equipped with a Leica DFC420 camera.

### Cytoplasmic and mitochondrial protein extraction

Cytoplasmic and mitochondrial proteins were extracted using a mitochondrial and cytoplasmic protein extraction kit (Beyotime, Jiangsu, China) according to themanufacturer’s instructions.

### Quantitative real-time PCR analysis

To measure target mRNA expression levels, total RNA was isolated from HL-7702 cells with Trizol (Invitrogen, Carlsbad, CA, USA) according to the manufacturer’s instructions. First-strand cDNA was synthesized using the RevertAid first-strand cDNA synthesis kit (Fermentas, Vilnius, Lithuania). PCR products were measured by real-time PCR with Ultra-Fast SybrGreen QPCR Master Mix (Agilent Technologies, Pala Alto, CA). The data were calculated using the following equation: relative mRNA expression = 2^−ΔΔCt^, where ΔΔCt = (Ct_sample_ − Ct_control_) treatment −(Ct_sample_ − Ct_control_) normal. The following primers were used: mortalin, 5′-CGCCCCACTTGTTTTGGA-3′ (sense) and 5′-CAGGAGTTGGTAGT ACCCAAATC-3′ (anti-sense); Mfn1, 5′-GCAACTGAAAAACTGAGGAT-3′ (sense) and 5′-ACTTGTTGGCACAGGCGAGC-3′ (anti-sense); Mfn2, 5′-ACTTGTTGGCA CAGGCGAGC-3′ (sense) and 5′-TGTTTGGCCGGGAGAGACGC-3′(anti-sense); Opa1, 5′-GCAGGCTCGTCTCAAGGATA-3′(sense) and 5′-TCTTCCAGTATAACC ACCTC-3′(anti- sense); hFis1, 5′-AAAGGGAGCAAGGAGGAACA-3′(sense) and 5′-TGGGGCTCTGTCTGCAGCAA-3′(anti-sense); Drp1, 5′-GATGGGAAGGGTTA TTGGAG-3′(sense) and 5′-CAGTTACACTCTTCTTGTTG-3′(anti-sense); MTP18, 5′-TCATGTCAGAGCCGCAGCCG-3′(sense) and 5′-TGGCACAAGAGAGCGGA AAG-3′(anti-sense);GAPDH, 5′-TTGCCATCAATGACCCCTTCA-3′(sense) and 5′-CGCCCCACTTGATTTTGGA-3′(anti-sense).

### Quantification of mitochondrial morphology

Quantitative analysis of mitochondrial shape and number was performed as previously described [HY, mito-analysis, mito-analysis3]. HL-7702 cells were stained with mitochondrial tracker green (MTG), a mitochondrial-specific fluorescent probe, and visualized using a fluorescence microscope equipped with a Leica DFC420 camera. Using ImageJ version 1.44, images were optimized by adjusting the contrast to span the complete gray range from 0 to 255, subjected to a 7 × 7 “top hat” spatial filter and thresholded. The 8-bit binary images were used for automated image analysis yielding the number of mitochondrial per cell (Nc), mitochondrial aspect ratio (AR: ratio between major and minor axes of the ellipse equivalent to the mitochondrion), and mitochondrial form factor (FF: perimeter^2^/4π * area). AR measured mitochondrial length, and FF determined mitochondrial length and degree of branching.

### Western blot analysis

Cells were lysed in SDS buffer supplemented with a mixture of protease inhibitors, 1 μg/ml aprotinin and 100 μg/ml phenylmethylsulfonyl fluorides. The cell suspension was incubated on ice for 30 min then centrifuged at 20,000 × g for 15 min at 4 °C. The supernatant was collected for further analysis. The protein concentrations of the supernatants were determined using the Bradford method. Cell lysates were denatured for 15 min in 5 × sample buffer and separated by SDS-polyacrylamide gel electrophoresis. For Western blot analysis, the gel was transferred onto nitrocellulose membranes using a tank transfer system. Blotted membranes were placed in a blocking solution of 5% non-fat milk in Tris-buffered saline Tween-20 (TBS-T). For immunodetection, membranes were incubated for 1 h at room temperature and then incubated overnight at 4 °C with the relevant primary antibodies. Then, the membranes were washed with TBST and incubated with the appropriate horseradish peroxidase-conjugated secondary antibodies. Immunocomplexes were visualized using a commercially available enhanced chemiluminescence kit with exposure of the transfer membrane to X-ray film. The following antibodies were used: anti-Bcl-2, anti-Bax, anti-Cyto C, anti-mortalin, anti-caspase 3, anti-β actin, and anti-glyceraldehyde-3-phosphate dehydrogenase (GAPDH).

### Primary hepatocyte isolation

Mouse primary hepatocytes were isolated from C57BL/6 aged 8–12 weeks by collagenase digestion and subsequent purification centrifugation fresh prepared hepatocytes were seeded in 6-well plates in attachment media. Cell media were cultured in DMEM supplemented with 10% fetal bovine serum, 100 U/ml penicillin and 100 U/ml streptomycin after 24 h. All animals received humane care and the experimental protocol was approved by the committee of laboratory animals according to institutional guidelines.

### Statistical analysis

Data were expressed as the means ± SEM from at least three independent experiments. Statistical significance analysis was performed by GraphPad Prism 5.0 software (GraphPad Software, Inc., La Jolla, CA) using Student’s t-test or ANOVA. Mean values were considered to be statistically significant at P < 0.05 or P < 0.01.

## Results

### The protective effects of SalB on H_2_O_2_-induced mitochondrial deformation

Mitochondrial morphology is crucial for proper cell function and physiology. Mitochondria are organized into reticular networks that undergo frequent shape changes. The mitochondrial shape is tightly controlled by the processes of mitochondrial fission-fusion^23^. However, the destruction of mitochondrial fission-fusion balance results in mitochondrial dysfunction and deformation and is associated with numerous human diseases^24^,^25^. To determine the effects of SalB on mitochondrial morphology, hepatocytes were stained with MTG, a mitochondrial-specific fluorescent probe, and observed by fluorescence microscopy. Quantitative analyses of the mitochondrial morphology were reported previously. Important mitochondrial morphological parameters, including AR (aspect ratio) that reflects mitochondrial length and FF (form factor) and represents mitochondrial length and degree, were analyzed using Image J Software I. In order to determine the proper working concentrations of H_2_O_2_, we measured the cell viabilities of HL-7702 cells. HL-7702 cells were treated with H_2_O_2_ (200, 400, 800, 1200, 1600, 2000, 2400, 2800 or 3200 μM) respectively for different time points (1 h, 2 h, 3 h or 4 h). Results showed (Supplementary Figure 1) thatHL-7702 cells treated with H_2_O_2_ (400 μM) for 1 h or 2 h, the cell viability decreased about 50% compared to the non-treated group. So we selected 400 μM and 2 h to perform the following experiments.

**Figure 1:**
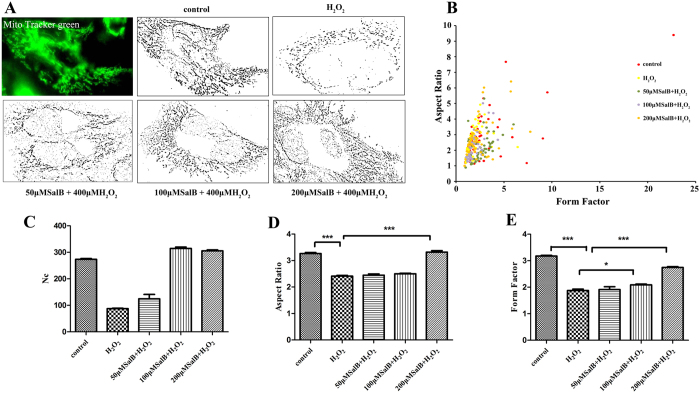
SalB inhibit mitochondrial deformation induced by H_2_O_2_. (**A**) Mitochondria from HL-7702 cells in the presence or absence of SalB were labeled with the mitochondrial-specific dye MTG for 30 min at 37 °C and viewed by fluorescence microscopy. Cells grown in normal medium exhibit long, tubular mitochondria; cells treated with H_2_O_2_ exhibit short, fragment mitochondria. SalB prevent H_2_O_2_-induced fragmentation and exhibit long, tubular mitochondria. (**B**) The graph depicts FF versus AR values for individual mitochondrion. Mitochondria of cells grown in normal medium have increased FF and AR values corresponding to long, tubular mitochondria. Fragmented mitochondria have reduced FF and AR values, n = 50 mitochondria. (**C**) The mitochondrial number of cells grown in H_2_O_2_ is significantly decreased compared with normal cells. (**D**,**E**) As mitochondrial morphological parameters, AR and FF were quantified by Image software. Images from a total of 15 individual cells were analyzed through three independent experiments. *P < 0.05 versus H_2_O_2_-treated control.

Our results revealed that mitochondrial morphology of hepatocytes was mostly tubular in the control group upon exposure to 400 μM H_2_O_2_ for 2 h. Considerably truncated and smaller mitochondria were prevalent in hepatocytes, indicating mitochondrial fragmentation. However, pretreatment with 50, 100, and 200 μM SalB suppressed the mitochondrial fragmentation and maintained normal mitochondrial tubular structure (Fig. 1A). For objective quantification of mitochondrial morphology, we analyzed mitochondrial shape by computer-assisted morphometric analyses that calculate AR and FF. AR and FF values increased as mitochondria elongate. Our data indicated that most mitochondria from cells treated with 400 μM H_2_O_2_ for 2 h had lower FF and AR values compared with the control. However, pretreatment with SalB inhibited the reduction in AR and FF caused by H_2_O_2_-induced oxidative stress (Fig. 1B–D).

### SalB protects against mitochondrial dysfunction induced by H_2_O_2_

**Figure 2:**
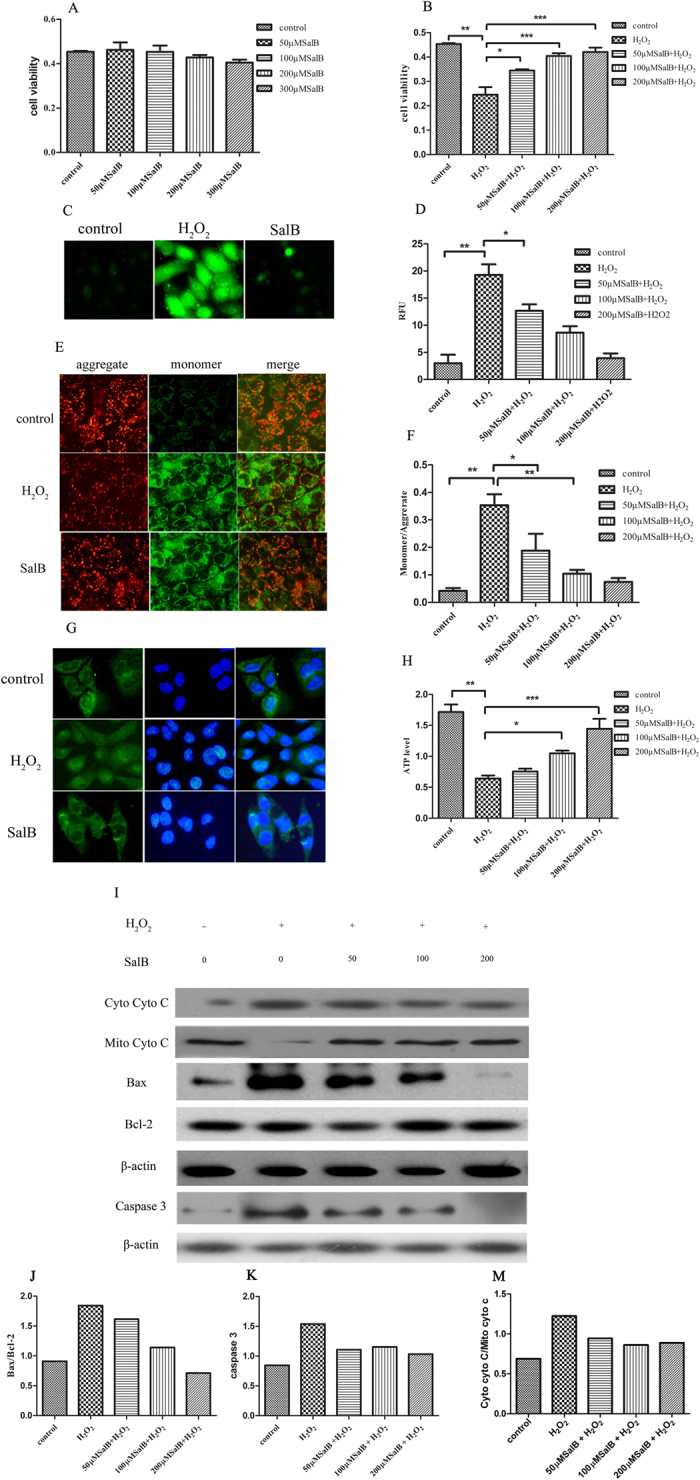
Effects of SalB on H_2_O_2_-induced mitochondrial dysfunction. (**A**,**B**) Cells were treated without or with SalB (50, 100, 200 μM) for 2 h followed by incubation with 400 μM H_2_O_2_ for an additional 2 h. After this incubation, cell viability was determined using the MTT assay. Data are expressed as the percentage (%) of the control values as the means ± SEM (n = 6) of representative experiments. (**C**,**D**) ROS of 7702 cells pre-incubated by 200 μMSalB for 2 h in the absence or presence of H_2_O_2_ (400 μM) were evaluated by the oxidation of H2DCF-DA to DCF. Intracellular ROS were determined by fluorescence microscopy (20×) and flow cytometry. (**E**,**F**) Mitochondrial membrane potential was detected by fluorescence microscopy (20×) and flow cytometry using JC-1. (**G**) Immunofluorescence analyses of cellular cytochrome c location as imaged by fluorescence microscope (20×).(**H**) Cellular ATP levels were measured using firefly luciferase. (**I**) Immunoblot shows mitochondrial cytochrome c release to the cytosol induced by H_2_O_2_. However, SalB effectively inhibited cytochrome c release from mitochondria. Immunoblot with cell extracts from 7702 cells pretreated with SalB for 2 h prior to exposure to H_2_O_2_. The blot shows Bax, Bcl-2 and caspase 3 protein expression levels. The results are expressed as the means ± SEM from three independent experiments (*P < 0.05; **P < 0.01; ***P < 0.001). (**J**,**K**) Quantitative results from Western blots. β-actin was used as a loading control.

Mitochondria are main intracellular organelles related to apoptosis. Thus, it is interesting to investigate the potential anti-oxidative effect of SalB on mitochondrial function^26^. HL-7702 hepatocytes were used to confirm the protective effects of SalB on hepatocyte injury induced by oxidative stress. Cell viability was not altered in cells treated with various concentrations of SalB for 2 h, suggesting that (50–200 μM) SalB is not cytotoxic to cells (Fig. 2A). Pretreatment with (50–200 μM) SalB for 2 h resulted in dose-dependent cytoprotective effects on cell viability against the damage caused by H_2_O_2_ (Fig. 2B). Intracellular ROS were measured with DCFH-DA upon exposure to 400 μM H_2_O_2_ for 2 h. A significant increase in ROS was noted compared with the control group. Then, pretreatment with SalB markedly prevented ROS accumulation in a concentration-dependent manner (Fig. 2C,D). MMP levels were measured using JC-1, which exists as a mitochondrial aggregate with red fluorescence or as cytoplasmic monomers at low mitochondrial potential resulting in green fluorescence. Thus, the monomer/aggregate ratio is used to monitor changes in MMP. As shown in Fig. 2E,F, cells exposed to 400 μM H_2_O_2_ for 2 h markedly downgraded MMP levels, which are related to mitochondrial dysfunction. However, SalB significantly improved the impairment of MMP. Mitochondrial membrane integrity is essential for ATP production, the disruption of MMP coupled with ATP depletion, and mitochondrial cytochrome c release, which promote an increase in mitochondrial apoptosis-related factors^27^. Therefore, ATP, cytochrome c, Bax, Bcl-2 and caspases were monitored. Intracellular ATP was detected by luciferase. SalB blocked the decrease of ATP content induced by H_2_O_2_ in a dose-independent manner (Fig. 2H). Next, we investigated the effect of SalB on H_2_O_2_-induced cytochrome c release from mitochondria to the cytosol (Fig. 2G). SalB markedly prevented the release of cytochrome c. Bcl-2 family members are major regulators of mitochondrial integrity, mitochondrial-initiated cytochrome c release and caspase activation^28^. SalB suppressed caspase activation under H_2_O_2_-induced stress conditions and inhibited the augmentation of Bax protein levels related to the mitochondrial apoptotic pathway (Fig. 2I–O).

### SalB mediated the up-regulation of mortalin in hepatocytes

**Figure 3:**
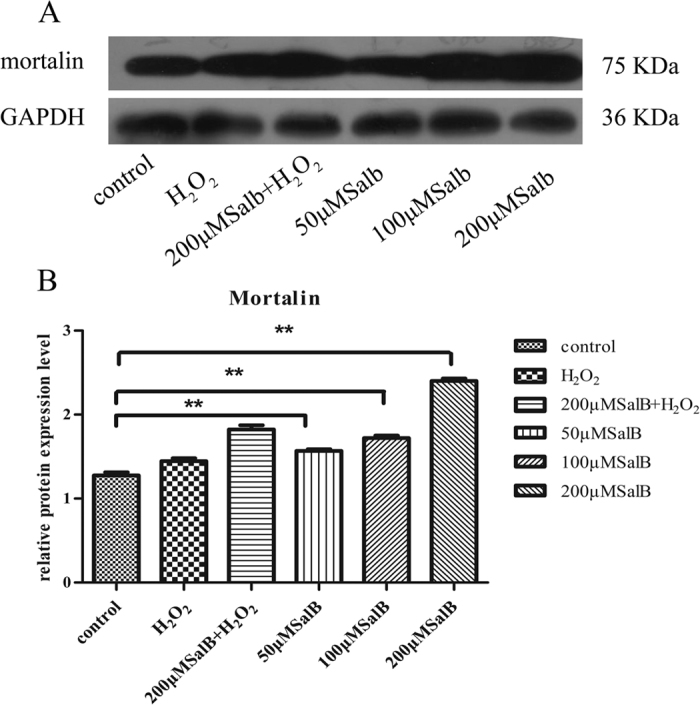
SalB up-regulates mortalin protein expression. (**A**) Immunoblot analysis. SalB increased mortalin expression levels compared with the control. GAPDH was used to normalize protein loading. A representative image of three experiments is presented. Full-length blots are presented in Supplementary Figure 2. (**B**) Quantity one software analysis of mortalin protein levels. Data are presented as the means ± SEM (n = 3, *P < 0.05).

The results suggested that SalB protects against mitochondrial dysfunction and maintains normal mitochondrial morphology. However, the mechanism of action of SalB on mitochondrial protection has not been thoroughly elucidated. As a central mitochondrial molecular chaperone, mortalin plays an important role in maintaining mitochondrial function. Mortalin prevents mitochondrial damage by eliminating ROS during glucose deprivation, and mortalin knockdown induces mitochondrial fragmented in HeLa cells^20^,^22^. Therefore, we assessed whether the protective effects of SalB on mitochondrial function and morphology are mediated by mortalin. To assess the effects of SalB on mortalin protein levels, hepatocytes were pre-incubated with different concentrations of SalB (50–200 μM) for 2 h. Pre-treatment with SalB (100 or 200 μM) significantly increased mortalin protein expression levels (Fig. 3A,B).

### Mortalin knockdown inhibits the protective effects of SalB on mitochondria

**Figure 4:**
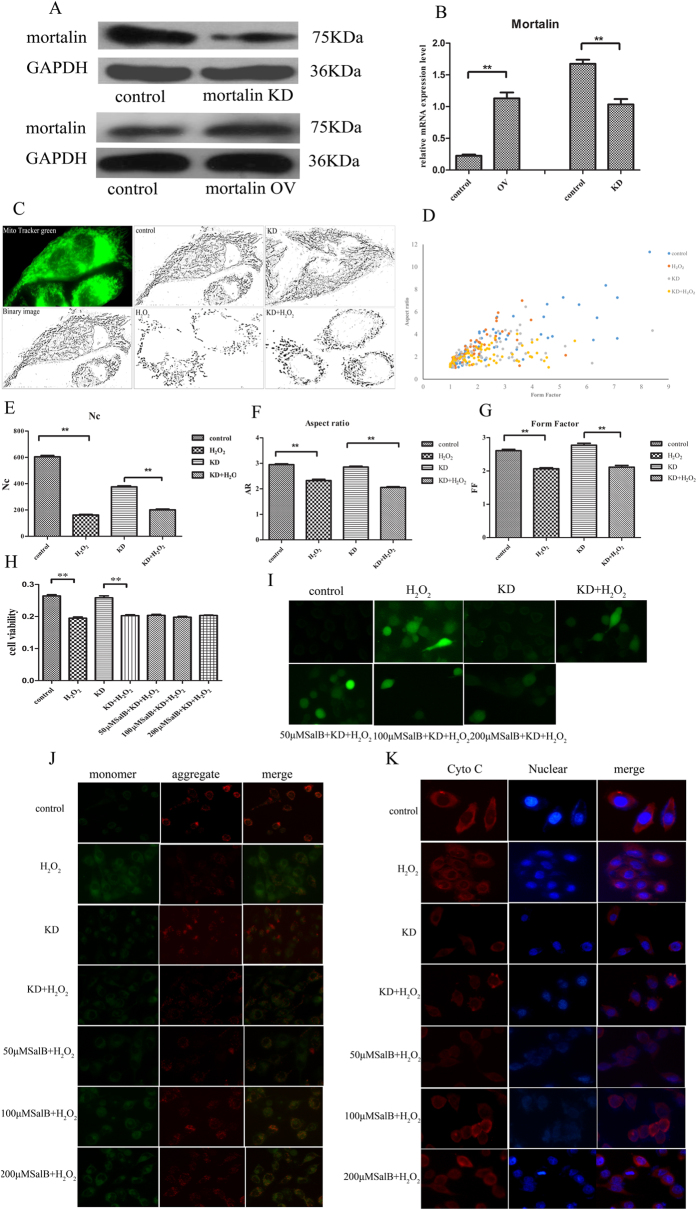
Mortalin knockdown blocks the protective effects of SalB on mitochondria. (**A**) Cells were infected with lentivirus shRNA and transfected with a mortalin-overexpression plasmid. Mortalin protein levels in the knockdown group were remarkably decreased, and mortalin protein levels in the overexpression group were increased. GAPDH was used as an internal control to normalize protein loading. Full-length blots are presented in Supplementary Figure 3. (**B**) Real-time PCR analysis. Mortalin mRNA expression levels in the knockdown group were significantly decreased, and mortalin mRNA expression in the overexpression group was notably increased (n = 3, ***P < 0.001). Data are normalized as a percentage of the control group and represented as the means ± SEM. (**C**) Mitochondria from control and mortalin knockdown 7702 cells were labeled with the mitochondrial-specific dye MTG for 30 min at 37 °C and viewed with a fluorescence microscope. Cells grown in normal medium exhibit long, tubular mitochondria. Cells treated with H_2_O_2_ exhibit short, fragmented mitochondria. Mortalin knockdown promotes mitochondrial truncation and fragmentation under H_2_O_2_ treatment. (**D**) The number of mitochondria in cells grown in H_2_O_2_ is significantly decreased compared with normal cells; mortalin knockdown decrease Nc with or without H_2_O_2_. (**E**,**F**) As mitochondrial morphological parameters, AR and FF were quantified by ImageJ software. Images from total 15 individual cells were analyzed through three independent experiments. *P < 0.05 versus H_2_O_2_-treated control. (**G**) The graph depicts FF and AR values for individual mitochondrion. Mitochondria of cells grown in normal medium have increased FF and AR values corresponding to long, tubular mitochondria. Fragmented mitochondria have reduced FF and AR values. n = 50 mitochondria. (**H**) Mortalin-knockdown cells were treated without or with SalB (50, 100, 200 μM) for 2 h followed by treatment with 400 μM H_2_O_2_ for an additional 2 h. After this incubation, cell viability was determined by the MTT assay. Data are expressed as the percentage (%) of control values, which are means ± SEM (n = 6) of representative experiments. (**I**) Cellular ROS were measured by a fluorescence microscope using H2DCF-DA dye. (**J**) Mitochondrial membrane potential was determined using JC-1 dye. (**K**) Immunofluorescence analysis of cellular cytochrome c location.

To further investigate the effects of mortalin on mitochondrial shape, human mortalin overexpression and knockdown plasmids were constructed. Hepatocytes were transfected with mortalin overexpression and knockdown plasmids and assessed by real-time PCR and Western blot analyses. As shown in Fig. 4A, immunoblot analysis revealed that the overexpression plasmid notably increased mortalin expression, whereas the mortalin knockdown plasmid resulted in remarkable suppression of mortalin protein levels. The mortalin mRNA expression level in the overexpression group was markedly elevated compared with the control group. In the mortalin knockdown group, the mortalin mRNA was considerably reduced compared with the control (Fig. 4B).

Our data suggested that SalB mediated the increase in mortalin protein levels, we hypothesized that SalB prevented hepatocyte injury induced by oxidative stress, which up-regulates mortalin. To test this hypothesis, we transfected mortalin knockdown plasmids into HL-7702 hepatocytes. First, to determine the effects of mortalin knockdown on mitochondrial shape and function, we observed mitochondrial morphology using MTG as visualized by fluorescence microscopy. We found significant truncated and fragmented mitochondria in mortalin knockdown group under H_2_O_2_-induced stress conditions (Fig. 4C,D). Morphometric analysis revealed no obvious alterations in mitochondrial AR and FF in the control and mortalin-knockdown group. However, after treatment with 400 μM H_2_O_2_ for 2 h, the mortalin-knockdown group exhibited a significant decrease in elongation (Fig. 4F,G).

To demonstrate the role of SalB and mortalin knockdown in H_2_O_2_-induced mitochondrial dysfunction, cell viability, ROS, MMP and cytochrome c were monitored. Under normal conditions, the viability of mortalin knockdown cells and normal cells was approximately the same. Upon exposure to 400 μM H_2_O_2_ for 2 h, the mortalin-knockdown group had a significant decrease in cell viability. Pretreatment with different concentrations of SalB in mortalin-knockdown cells had no significant protective effects on cell viability under H_2_O_2_-induced stress conditions (Fig. 4H). We measured intracellular ROS levels and found that intracellular ROS levels markedly increased after treatment with H_2_O_2_ and incubation with SalB. SalB did not eliminate intracellular ROS accumulation induced by H_2_O_2_ (Fig. 4I). Next, upon exposure to 400 μM H_2_O_2_ for 2 h, the mortalin-knockdown group exhibited a significant decrease in MMP levels, and treatment with SalB did not alter MMP levels (Fig. 4J). In addition, we investigated the protective role of SalB on cytochrome c release in mortalin-knockdown hepatocytes induced by H_2_O_2_. SalB did not prevent the release of cytochrome c from mitochondria to the cytosol (Fig. 4K). These results suggested that SalB did not prevent mitochondrial dysfunction induced by H_2_O_2_ under conditions of mortalin knockdown.

### The effects of mortalin overexpression on mitochondrial fission and fusion

**Figure 5:**
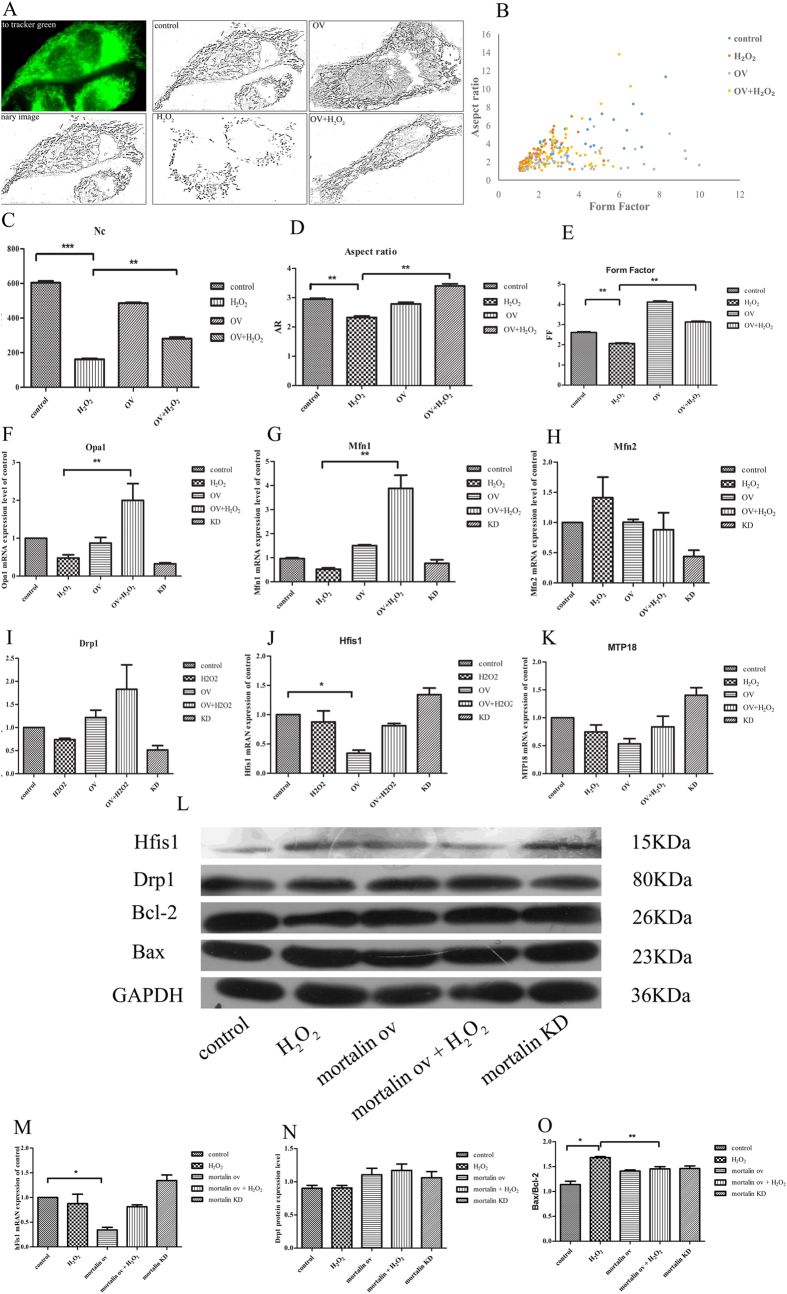
Effects of mortalin overexpression on mitochondrial fission and fusion. (**A**) Mitochondria from control and mortalin-overexpression HL-7702 cells were labeled with the mitochondrial-specific dye MTG for 30 min at 37 °C and viewed with a fluorescence microscope. Cells grown in normal medium exhibit long, tubular mitochondria. Cells treated with H_2_O_2_ exhibit short, fragmented mitochondria. Mortalin overexpression inhibited mitochondrial truncation and fragmentation under H_2_O_2_ treatment. (**B**) The number of mitochondria in cells grown in H_2_O_2_ is significantly decreased compared with normal cells. (**C**,**D**) As mitochondrial morphological parameters, AR and FF were quantified by ImageJ software. Images from 15 individual cells were analyzed inthree independent experiments. *P < 0.05 versus H_2_O_2_-treated control. (**E**) The graph presents FF and AR values for individual mitochondrion. Mitochondria of cells grown in normal medium have increased FF and AR values corresponding to long, tubular mitochondria. Fragmented mitochondria have reduced FF and AR values. n = 50 mitochondria. (**F**–**K**) Real time PCR was used to analysis mitochondrial fusion factors Mfn1, Mfn2, and Opa1 and mitochondrial fission factors hFis1, Drp1 and MTP18. These factors were normalized to GAPDH mRNA expression. Three independent experiments are presented. Data are normalized as a percentage of the control group and represent means ± SEM, *P < 0.05 versus control group, n = 3. (**L**) Western blot analysis of hFis1, Mfn2, Bax, Bcl-2 levels in HL-7702 cell treatment with different concentration of SalB, SalB increased hFis1 protein level and inhibited Mfn2 protein expression. Full-length blots are presented in Supplementary Figure 4. (**M**–**O**) Quantitative results from Western blots. GAPDH was used as a loading control (*P < 0.05; **P < 0.01).

We examined whether mortalin overexpression prevented mitochondrial fragmentation induced by H_2_O_2_ and how mortalin regulated mitochondrial morphology changes by mediating mitochondrial fission and fusion related factors. We investigated the roles of mortalin overexpression on H_2_O_2_-induced mitochondrial deformation; hepatocytes were transfected with mortalin overexpression plasmids. Our studies demonstrated that mortalin overexpression obviously improved the formation of mitochondrial network structure compared with the control group. After treatment with 400 μM H_2_O_2_ for 2 h, cells with mortalin overexpression maintained a normal mitochondrial network structure (Fig. 5A). The two parameters AR and FF were used for objective quantification of mitochondrial shape. As shown in Fig. 5D,E, upon exposure to 400 μM H_2_O_2_ for 2 h, the mortalin overexpression group markedly exhibited increased AR and FF values compared with the H_2_O_2_-induced group. These results indicated that mortalin overexpression prevented the changes of mitochondrial deformation induced by H_2_O_2_. How mortalin regulated the alterations of mitochondrial shape remains unknown. Mitochondrial morphology and number continually change in the living cell and are predominantly maintained by the balance of fission and fusion^29^,^30^. Mitochondrial fission is primarily mediated by dynamic related protein 1 (Drp1), human fission 1 (hFis1) and mitochondrial protein 18 (MTP18). In contrast, mitochondrial fusion is executed by Mitofusin 1 and 2 (Mfn1 and Mfn2) and Optic atrophy 1 (Opa1)^31^,^32^. To determine whether mortalin overexpression affects mRNA levels of fusion and fission-related factors, real-time PCR analysis was performed. As shown in Fig. 4B, in normal conditions, mortalin overexpression reduced hFis1 mRNA expression levels but did not affectDrp1, MTP18, Opa1, Mfn1 and Mfn2 mRNA expression. The results indicated that mortalin overexpression promoted mitochondrial network structure by down-regulating hFis1 mRNA levels to reduce mitochondrial fission. Upon exposure to 400 μM H_2_O_2_, mortalin overexpression increased Opa1 and Mfn1 mRNA expression but did not affect the mRNA expression level of other factors (Fig. 5F–K). This result suggested that mortalin inhibit H_2_O_2_-induced mitochondrial division by up-regulating mitofusin-related factors Opa1 and Mfn1 to enhance mitochondrial fusion. At the same time SalB increased hFis1 protein level (Fig. 5L–O), which indicated mortalin maintain mitochondrial structure stabilization by up-regulating mitofusin factor Mfn2 and down-regulating mitofission hFis1.

### SalB balances mitochondrial fusion-fission through up-regulate mortalin in primary hepatocyte

**Figure 6:**
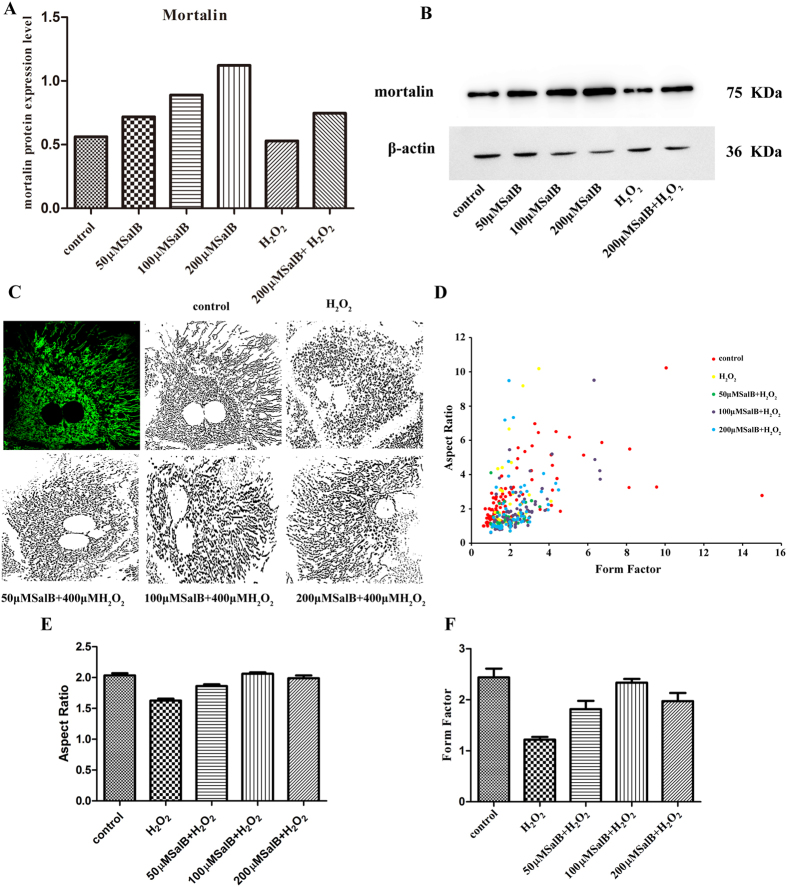
SalB banlances mitochondrial fusion-fission through up-regulate mortalin in primary hepatocyte. (**A**) Western blot analysis showed the expression of mortalin was higher in SalB treated groups compare to control group. β-actin was used to normalize protein loading. The representative of three experiments was shown. Full-length blots are presented in Supplementary Figure 5. (**B**) Quantitative results from Western blots (n = 3, *P < 0.05). (**C**) Confocal fluorescence microscope (60×) images showed that treatment with H_2_O_2_ induced mitochondrial fission, while pretreatment with 50, 100, 200 μM SalB can inhibit mitochondrial structure instability induced by H_2_O_2_. (**D**) The values of FF versus AR for individual mitochondrion, n = 50. (**E**,**F**) AR and FF were mitochondrial morphological parameters, treatment with SalB augmented AR and FF values, which means increased number of long tubular mitochondria. Images from a total of 15 individual cells were analyzed through three independent experiments, *P < 0.05.

Our previous results showed that SalB could preserve the morphological and functional integrity of mitochondriaby up-regulating mortalin in hepatocyte cell line HL-7702. In order to further explore the effects of SalB on mortalin expression in primary hepatocyte, we incubated primary hepatocyte with 50, 100, 200 μM SalB for 2 h. Western blots showed that SalB significantly increased mortalin in primary hepatocyte (Fig. 6A and B). At the same time, a decrease in mortalin protein level was observed in H_2_O_2_ (400 μM) treated group, however pretreatment with 200 μM SalB restored mortalin expression. We further examined the effects of SalB on mitochondrial morphology in primary hepatocyte. Confocal fluorescence microscope images showed (Fig. 6C) that mitochondrial morphology in primary hepatocytes were long and tubular, however in H_2_O_2_ treated group, truncated and smaller mitochondria increased. And truncated and smaller mitochondria indicate mitochondrial fragmentation. Pretreatment with SalB (50, 100 and 200 μM) for 2 h prohibited mitochondrial fragmentation. Aspect ratio and Form factor were two mitochondrial morphological parameters, which reflect mitochondrial status. Our data (Fig. 6D–F) showed that incubation with 200 μM H_2_O_2_ significantly reduced the values of AR and FF, which means more fragmented, smaller and truncated mitochondrial generation, primary hepatocytes’ mitochondrial fission more quick than fusion. However, pre-incubation with SalB (50, 100 and 200 μM) for 2 h increase the values of AR and FF, appearing more tubular and long structure mitochondrial. From all these results, we suppose that SalB can preserve the mitochondrial morphology by up-regulating mortalin in primary hepatocytes.

## Discussion

One of the initial causes of hepatic fibrosis is an increased number of hepatocyte apoptosis, which is a main factor contributing to the development and progression of liver diseases^33^,^34^. The first goal of our research was to determine the effects of SalB on the mitochondrial pathway. Cell apoptosis is mainly transduced through the following two defined pathways: the mitochondrial apoptotic pathway and death receptor pathway. The mitochondrial pathway plays a critical role in hepatocyte apoptosis^9^. Our data showed that H_2_O_2_-induced oxidative stress leads to a significant decrease in cell viability, abnormal accumulation of ROS, MMP collapse, ATP depletion and mitochondrial cytochrome c release from mitochondria to the cytosol. In addition, oxidative stress activates caspase3 and increases the ratio of Bax/Bcl-2. However, pretreatment with SalB remarkably scavenges free radicals, maintains mitochondrial membrane permeability, guarantees energy basal metabolism, and inhibits the activation of apoptotic-related factors, indicating that the antioxidant properties of SalB notably rescue the mitochondrial dysfunction caused by H_2_O_2_-induced oxidative stress, which is consistent with other reports^3^,^35^,^36^. The morphology, interconnectivity and integrity of mitochondria play a critical role in regulating various aspects of mitochondrial functions, including ATP generation, mitochondrial DNA inheritance, Ca^2+^ buffering, free radical homeostasis and quality control^37^. Mitochondrial fission and fusion mediates mitochondrial morphology changes, which have become the focus of attention based on cells participating in apoptosis^38^,^39^,^40^. Mitochondrial division is a key step in apoptosis induced by severe oxidative stress^41^. We found that mitochondria are fragmented and truncated when subject to 400 μM H_2_O_2_-induced oxidative stress conditions for 0.5, 1, or 2 h via a mitochondrial division process (data not shown). The phenomenon is consistent with a previous study that reported that H_2_O_2_-induced oxidative stress caused mitochondrial fragmentation in C_2_C_12_ myocytes^42^. SalB prevents mitochondrial dysfunction by scavenging ROS. Our data demonstrate that pretreatment with SalB in hepatocytes prohibits mitochondrial fragmentation by decreasing mitochondrial fission under oxidative stress conditions. Thus, we ask the following question: does SalB maintain normal mitochondrial function by restraining mitochondrial fragmentation? These results indicate that SalB prevents H_2_O_2_-induced oxidative stress by regulating mitochondrial morphology and function.

Mortalin is involved in the maintenance of mitochondrial homeostasis and serves as a critical molecular chaperone. As an anti-apoptotic protein, mortalin overexpression protects against apoptosis by preventing the accumulation of redundant ROS in mitochondria, which are induced by glucose deprivation; chemical reperfusion in neuronal cells^20^; and iron chelating in a hepatocyte cell line^43^. Previous studies demonstrate that SalB and mortalin provide protective effects against mitochondrial dysfunction induced by oxidative damage. Therefore, we assume that SalB protects mitochondria via mechanisms mediated by mortalin. In our study, we found that pretreatment with (100 or 200 μM) SalB, significantly increased mortalin protein levels comparedwith the control group. The result indicates that SalB attenuates mitochondrial dysfunction by up-regulating mortalin.

Although previous reports demonstrate that mortalin provides effective protection against mitochondrial dysfunction, its role in relation to mitochondrial shape must be addressed. In our research, we reported that the effects of mortalin overexpression and knockdown on mitochondrial shape are altered in hepatocytes. Under normal conditions, mortalin overexpression strengthens mitochondrial network structure compared with the control group, but mortalin knockdown had no significant change in mitochondrial morphology compared with untransfected cells. The result is consistent with a recent study performed under normal conditions. Knockdown of endogenous mortalin did not cause a significant change in mitochondrial morphology^22^. Under oxidative stress conditions, mortalin overexpression inhibited mitochondrial fragmentation. However, mortalin knockdown failed to rescue mitochondria, which is similar to previous reports^22^. Our study suggests that mortalin overexpression protects against mitochondrial division induced by oxidative damage. In addition, in mortalin-knockdown hepatocytes, SalB did not prevent mitochondrial dysfunction induced by H_2_O_2_. Specifically, cell viability, ROS accumulation, MMP collapse and cytochrome c release were not altered. Therefore, it is reasonable to conclude that SalB maintains mitochondrial morphology and function by up-regulating mortalin in hepatocytes. Thus, we ask, how is mortalin involved in regulating mitochondrial structure? Mitochondrial shape change is mediated by mitochondrial fission and fusion. Mitochondrial fission is controlled by Drp1, hFis1 and MTP18, whereas mitochondrial fusion is mediated by Opa1, Mfn1 and Mfn2^23^,^32^. To investigate how mortalin regulates mitochondrial morphology, we studied mortalin interactions with fusion- and fusion-related factors. Under normal conditions, mortalin overexpression decrease hFis1 mRNA levels, which is involved in mitochondrial fission. Under oxidative stress conditions, mortalin overexpression increased Opa1 and Mfn1 mRNA levels, which are involved in mitochondrial fusion. These results suggest that under normal conditions, mortalin overexpression inhibit mitochondrial fragmentation by lowering mitochondrial fission, which is mediated by hFis1 protein. In contrast, mortalin overexpression prevents oxidative damage induced by the generation of numerous smaller and truncated mitochondria by enhancing mitochondrial fusion and reducing mitochondrial division, which is mediated by Opa1 and Mfn1. These data are similar with previous reports (Fig. 5F,G).

We also examined the effects of SalB on mortalin in primary hepatocytes, and the similar results were obtained. Pretreatment with SalB suppressed the mitochondrial deformation induced by H_2_O_2_ in primary hepatocytes. At the same time, mortalin was up-regulated by SalB in primary hepatocytes.

In conclusion, our data demonstrated that SalB prevents mitochondrial dysfunction and mitochondrial fragmentation from H_2_O_2_-induced oxidative damage by up-regulating mortalin. Importantly, we demonstrated that mortalin overexpression mediates the protein levels of fission-related factor of hFis1 and the mRNA levels of fusion-related factors of Mfn1 and Opa1 to maintain the dynamic balance of fission and fusion. Based on our data that SalB protects against hepatocyte apoptosis by up-regulating mortalin, mortalin can be a novel target to develop more traditional Chinese medicines for liver disease therapy.

However, our experiments were limited to the mechanism of action for SalB. Further studies are needed to confirm the interaction of SalB and mortalin and determine how SalB mediates mitochondrial fission and fusion-related factors to protect mitochondrial morphology *in vivo* experiments.

## Additional Information

**How to cite this article:** Liu, Y. *et al*. Salvianolic acid B inhibits mitochondrial dysfunction by up-regulating mortalin. *Sci. Rep.* **7**, 43097; doi: 10.1038/srep43097 (2017).

**Publisher's note:** Springer Nature remains neutral with regard to jurisdictional claims in published maps and institutional affiliations.

## Supplementary Material

Supplementary Information
