# Supplementary material for: Salvianolic acid B inhibits mitochondrial dysfunction by up-regulating mortalin
Source: Sci Rep. 2017 Mar 2;7:43097. doi: 10.1038/srep43097 (PMC5333085; doi:10.1038/srep43097)
Supplement: Supplementary Information [file srep43097-s1.pdf]

Salvianolic acid B inhibits mitochondrial dysfunction by up-regulating mortalin

Yunxia Liu[1,2,#], Yingying Hu [1,3,#], Qiukai E[1,4],Ji Zuo[1], Ling Yang[1,*], Wen Liu[1,*]

Supplementary Figure1

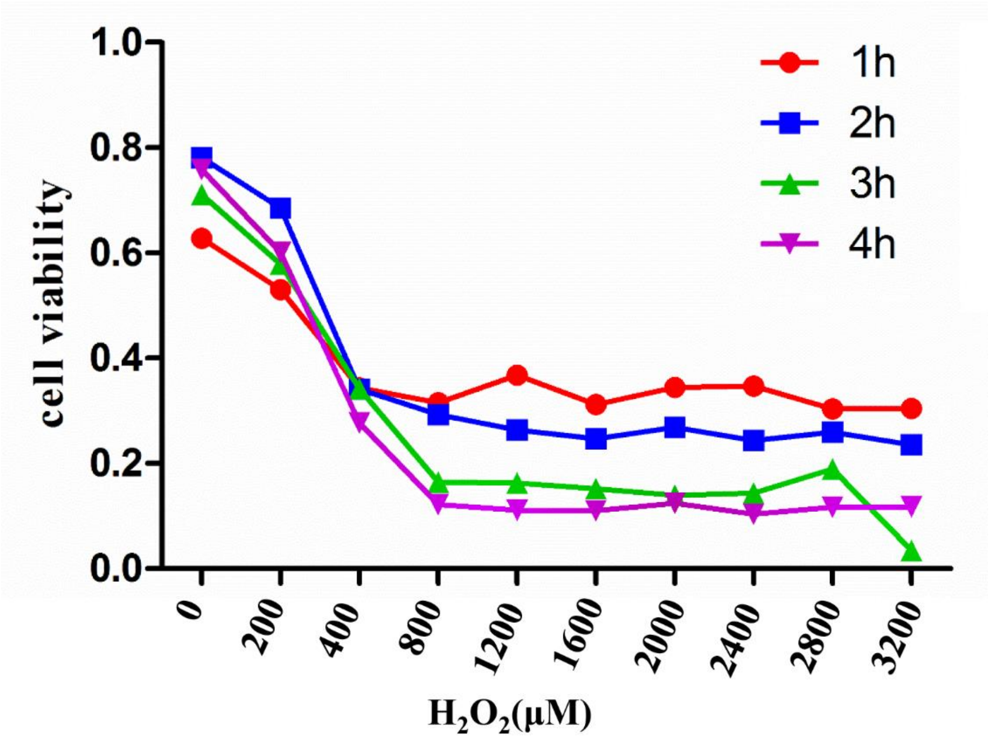

Supplementary Figure1 To determine the proper working concentrations of H_2O_2 through measuring the cell viabilities of HL-7702 cells. Cells were treated with H_2O_2 (200, 400, 800, 1200, 1600, 2000, 2400, 2800 or 3200 μM) respectively for different time points (1h, 2h, 3h or 4h).

Supplementary Figure 2

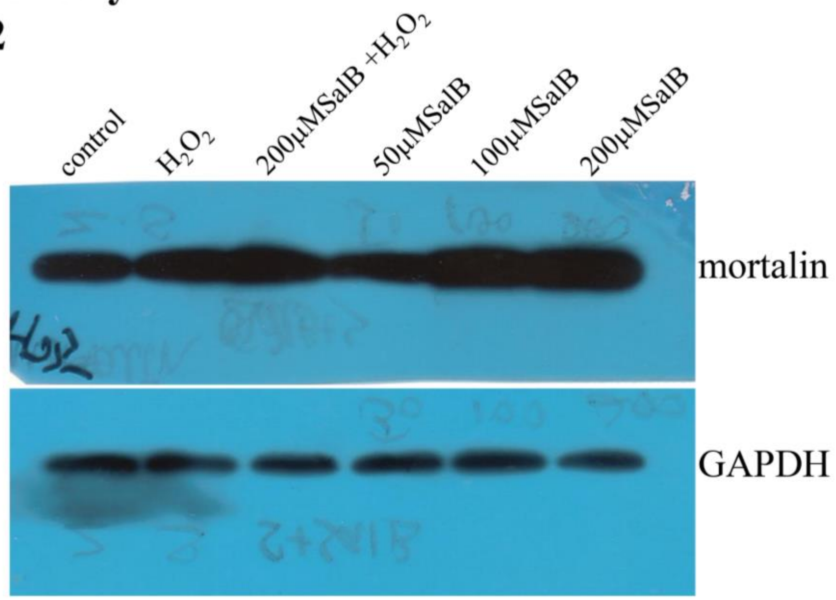

Supplementary Figure2 Full-length blots of Figure 3A.

Supplementary Figure 3

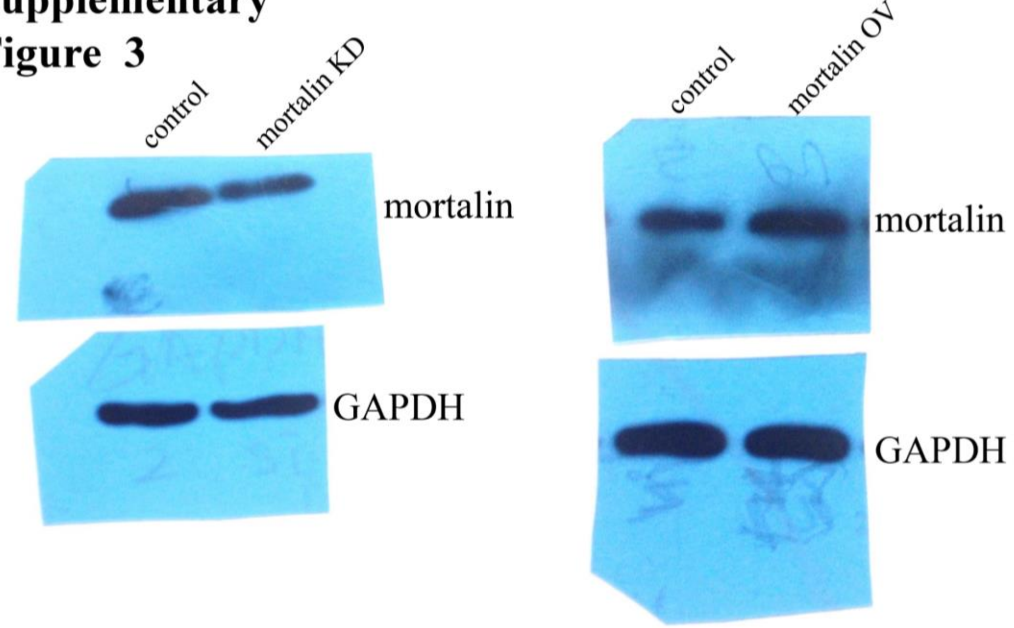

Supplementary Figure3 Full-length blots of Figure 4A.

Supplementary Figure 4

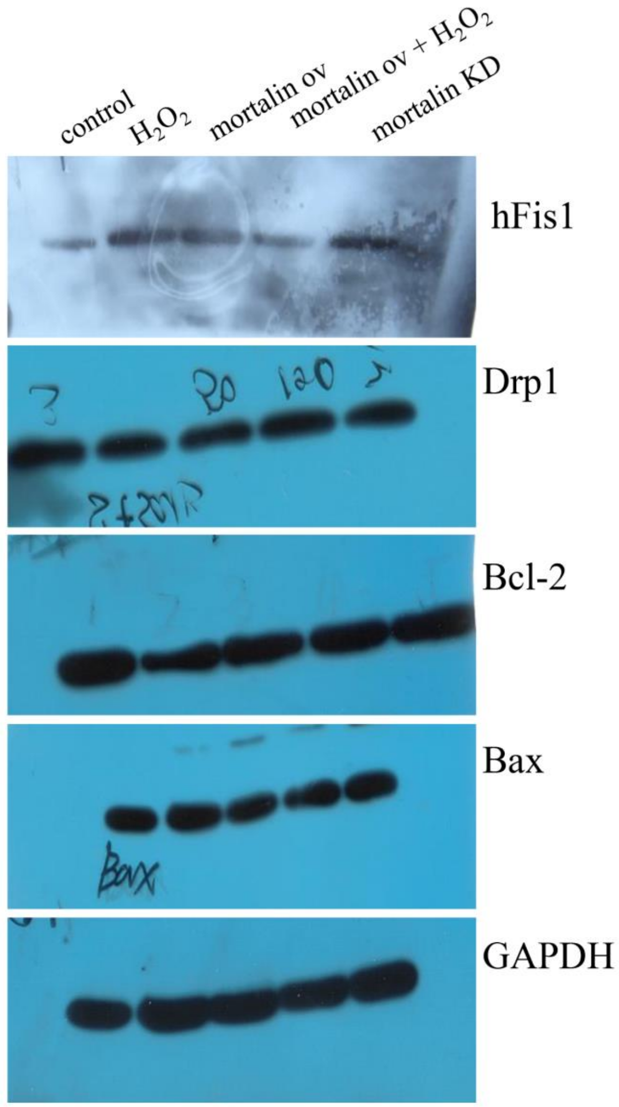

Supplementary Figure4 Full-length blots of Figure 5L.

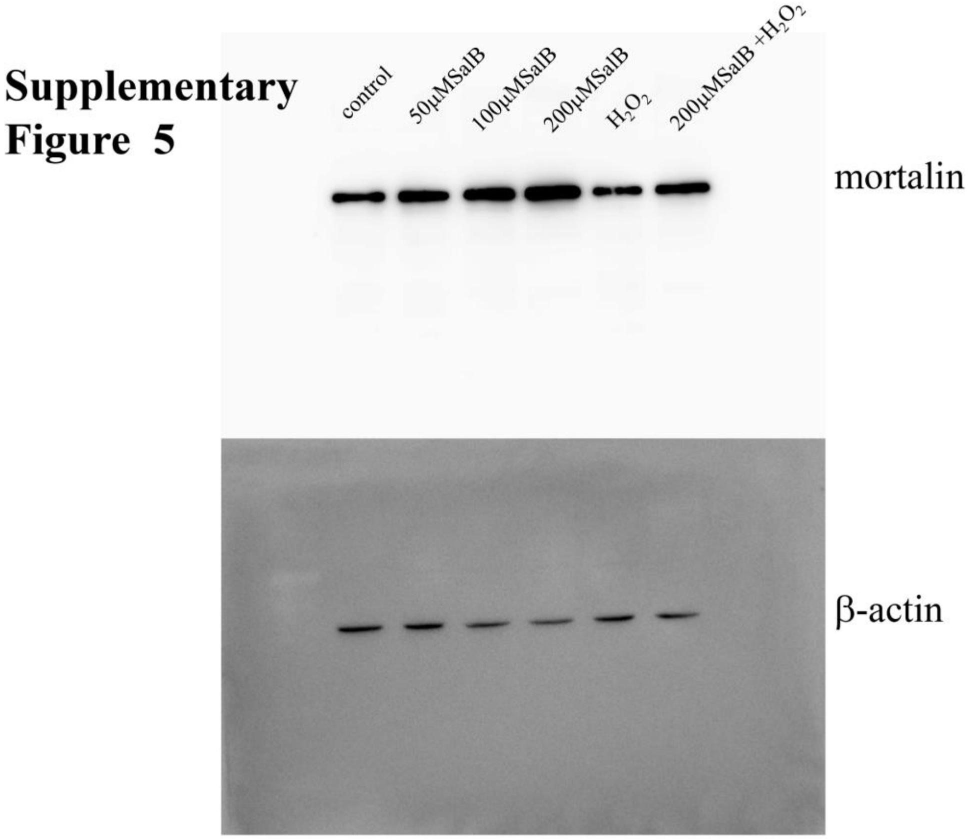

Supplementary Figure5 Full-length blots of Figure 6A.
